# Risk factors analysis and risk prediction model for failed back surgery syndrome: A prospective cohort study

**DOI:** 10.1016/j.heliyon.2024.e40607

**Published:** 2024-11-22

**Authors:** Parisa Hajilo, Behzad Imani, Shirdel Zandi, Ali Mehrafshan, Salman khazaei

**Affiliations:** a Student Operating Room (MSc), Student Research Committee, Hamadan University of Medical Sciences, Hamadan, Iran; b Department of Operating Room, School of Paramedicine, Hamadan University of Medical Sciences, Hamadan, Iran; c Department of Neurosurgery, Nekuii Forghani Hospital University of Medical Sciense Qom, Qom, Iran; d Hamadan University of Medical Sciences, Hamadan, Iran

**Keywords:** Risk factors, Prediction model, Surgery, Back, Syndrome, Failed

## Abstract

**Introduction:**

With the growing number of posterior open surgery, the incidence of failed back surgery syndrome (FBSS) increases gradually. Currently, there is a lack of predictive systems and scientific evaluation in clinical practice. This study aimed to risk factors analysis of FBSS and develop a risk prediction model.

**Materials and methods:**

Baseline data were collected from 512 patients. Patients were followed up for one year. Ultimately, 146 patients were classified in the FBSS group, with an incidence rate of 32.5 %. Logistic regression was used to screen for independent risk factors influencing the occurrence of FBSS. The diagnostic power of model was evaluated using the receiver operating characteristic (ROC) curve.

**Findings:**

Age, smoking, type of pain, revision surgery, surgical technique, quality of life, and psychological status were significantly associated with the incidence of FBSS. The strongest factor in this model was the selected surgical technique, with an odds ratio of 0.095. The area under the ROC curve for the model's diagnostic and classification power was 0.852.

**Conclusion:**

The causes of FBSS can stem from underlying factors, lifestyle, surgical causes, and patients' psychological factors. Therefore, prevention and treatment for each individual should be based on their specific cause to achieve optimal results.

## 1 Introduction

Degenerative lumbar disease (DLD) is recognized as the most common cause of low back pain, with its prevalence increasing with age [1]. The first line of treatment for these patients encompasses various non-surgical options, including lifestyle modifications, medications, and physiotherapy. However, surgical intervention is advised when symptoms persist. Over the past two decades, there has been a notable increase in the number of patients deemed suitable for spinal surgery [2]. Although initial structural defects are corrected post-surgery, some patients continue to experience persistent pain or limb numbness. A significant number of individuals suffer from chronic pain in the lower back and legs, along with ongoing functional limitations [3]. Consequently, those patients who fail to improve post-surgery are often classified with the heterogeneous disorder referred to as Failed Back Surgery Syndrome (FBSS). FBSS is defined as "a diverse and complex set of symptoms including persistent or recurrent chronic pain after one or more spinal surgeries" [4]. This term currently describes a heterogeneous group of patients whose surgical outcomes do not align with the pre-surgical expectations of both the patient and the surgeon [5]. Despite advancements in our understanding of anatomical structures and the expansion of minimally invasive techniques, the prevalence of FBSS is rising due to this multifaceted issue characterized by diverse underlying causes [6]. Multiple factors (biological, psychological, and social) are involved in the development of the pain process, necessitating an interdisciplinary approach to further clarify its causes. Researchers posit that factors such as age, lifestyle choices (smoking, obesity, inactivity), the presence of specific comorbidities, the severity of preoperative pain, and psychosocial aspects are potential characteristics associated with the incidence of FBSS [7]. Additionally, studies indicate that incorrect surgical techniques, surgical complications, instability, recurrent disc herniation, and neuropathic pain significantly impact the occurrence of FBSS [8]. Modern medical research has identified various complex factors involved in the FBSS process. However, managing pain requires a multidimensional approach, complicating the determination of the exact causes of FBSS [9]. Therefore, early screening and effective prevention of FBSS have become critical issues for healthcare professionals. In contemporary medicine, accurately predicting the occurrence and prognosis of diseases is increasingly important, as treatments should be individualized to achieve optimal outcomes. Expectations for outcomes should vary based on the type of structural problem, the number of previous surgeries, and the patient's mental health. Surgeons must convey realistic expectations to patients to align the expectations of both parties [10]. Numerous researchers have shown that developing risk prediction models can effectively reduce disease incidence [11,12]. Despite significant advances in diagnosing and treating FBSS, the lack of baseline epidemiological data and a scientific prediction system hinders successful evaluation of FBSS prevention and prognosis. Therefore, this study aims to identify the most critical risk factors and develop a robust risk prediction model for FBSS. The primary goal is to assist physicians and patients in enhancing prevention and treatment strategies for FBSS.

## 2 Materials and Methods

### 2.1 Study design

This study was conducted as a prospective cohort in Iran (Valiasr Hospital, Qom) in 2023 with a one-year follow-up. The research population included all patients who visited Valiasr Hospital for surgery due to degenerative lumbar disease (DLD) from January 2022 to April 2023. Patients were included in the study using available sampling method.The researcher enrolled all patients. The surgeries were performed by a single surgeon at a single center. The article's writing followed the Strengthening the Reporting of Observational Studies in Epidemiology (STROBE) checklist.

### 2.2 Inclusion and exclusion criteria

#### 2.2.1 Inclusion criteria

• Age between 20 and 60 years
• Patients with DLD
• Patients who can undergo magnetic resonance imaging (MRI) and computed tomography (CT) scans

#### 2.2.2 Exclusion criteria

• Patients diagnosed with specific diseases such as malignant tumors, vertebral fractures, spinal infections, inflammatory spondylitis
• Patients with progressive neurological deficits or severe concomitant neurological symptoms
• Patients with pain originating from non-spinal causes and/or soft tissue problems

### 2.3 Instruments and data collection

#### 2.3.1 Pre-operative

Demographic and clinical information forms for the patients were recorded through a questionnaire and an interview before surgery by the researcher. A detailed history was taken regarding the onset of pain, pain characteristics, pain location, pain pattern, and pain source.

Preoperative anxiety was assessed using the Barton et al. (2019) Surgical Anxiety Questionnaire. This questionnaire contains 27 items that measure preoperative anxiety across six dimensions. This questionnaire's minimum and maximum possible scores range from 19 to 95. A score below 38 indicates low surgical anxiety, a score between 39 and 76 indicates moderate anxiety and a score above 77 indicates high anxiety [13].

#### 2.3.2 During operative

##### 2.3.2.1 Surgical technique

Surgery was performed on all patients under general anesthesia in the prone position. A midline incision measuring 5–15 cm (depending on the type of surgery) was made. After muscle dissection, bilateral decompression (laminectomy, medial facetectomy, flavectomy, discectomy) was performed. Following decompression, for the Fixation and Fixation + PLIF (Posterior Lumbar Interbody Fusion) groups, pedicle screws were placed unilateral or bilateral (depending on the individual condition), and the pedicle screw anchoring process was completed. Then, in the Fixation + PLIF group, after preparing the endplates, an intervertebral cage was placed and fixed [14].

The amount of blood loss (Blood gases + bottle suction), the time of surgery (from skin incision to the last suture), the type of DLD (Degenerative disc disease, Spinal stenosis, Spinal instability, Spondylolisthesis, Spondylolysis), the type of surgical procedure (with fixation (unilateral, bilateral), without fixation, with and without interbody fusion), the number of surgical levels, and sacrum fixation were recorded by the researcher.

#### 2.3.3 Postoperative

Postoperative psychological disorders, quality of life, and sleep quality of the patients were assessed using validated questionnaires. The psychological disorders questionnaire (SCL-25) developed by Najarian and Davoudi (2001) consists of 25 questions. These questions assess various psychological conditions, including anxiety, depression, phobic anxiety, paranoid ideation, psychosis, obsessive-compulsive disorder, and somatic complaints. Scores in this questionnaire range from a minimum of 25 to a maximum of 125. Specifically, scores between 25 and 50 suggest the presence of low psychological disorders, scores from 50 to 75 indicate moderate psychological disorders, and scores above 75 reflect high psychological disorders [15].

Patients' quality of life was evaluated using the Short Form Health Survey (SF-36), which assesses eight health domains: physical function, role function-physical, bodily pain, general health, vitality, social function, role function-emotional, and mental health. The scores range from 0 to 100, where 0 indicates the lowest quality of life and 100 represents the highest [16].

The Pittsburgh Sleep Quality Index (PSQI) includes 19 items that evaluate sleep quality across seven scales: subjective sleep quality, sleep latency, sleep duration, habitual sleep efficiency, sleep disturbances, use of sleeping medication, and daytime dysfunction. The total score across these scales forms a global score ranging from 0 to 21, with a score of 6 or higher indicating poor sleep quality [17].

After a 12-month follow-up, the patients were divided into two groups. The first group (non-FBSS) consisted of patients who were satisfied with their surgery and had completely recovered. The second group (FBSS) included patients classified under the Failed Back Surgery Syndrome category. Various definitions of FBSS patients have been provided in similar studies, as outlined below. In FBSS patients, chronic radicular pain in the same area may recur or persist despite successful spinal surgery [4]. Additionally, the outcomes of lumbar spinal surgery may fail to meet the preoperative expectations of both the patient and the surgeon [1]. Patients are categorized as having chronic neuropathic pain if they experience pain for three months or longer, with a Visual Analog Scale (VAS) score higher than five in the lumbar area, with or without radiation to the limbs [5]. Moreover, these patients may experience chronic back or leg pain following successful lumbar surgery without identifiable causes such as compressive lesions or infections [8]. To ensure accurate diagnosis, a quantitative and systematic definition of FBSS was established: intractable pain or sensory deficit in the back and/or limbs post-surgery, resistant to conservative treatment for more than three months, resulting in dissatisfaction with the surgical outcome.

### 2.4 Statistical analysis

The sample size in this study was calculated using the Morgan table. We analyzed the data using SPSS version 16. First, we assessed the equality of variances and the assumption of normality using Levene's and the Shapiro-Wilk tests. Continuous data were analyzed with the independent *t*-test and presented as mean (SD). Categorical data were analyzed using the chi-square test and presented as numbers (%). The Omnibus test was used to evaluate the explanatory and predictive power of the logistic regression model. A binary logistic regression analysis was conducted to develop the FBSS risk prediction model, with FBSS as the dependent variable. The Receiver Operating Characteristic (ROC) curve and Area Under the Curve (AUC) were utilized to assess the diagnostic and classification power of the binary logistic regression model (univariate).

**Fig. 1:**
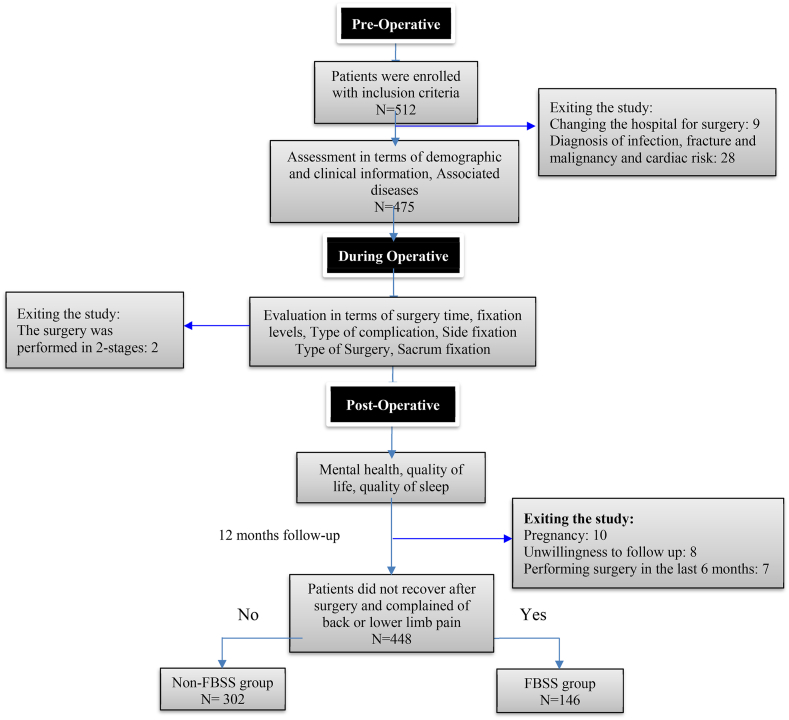
The STROBE flow diagram shows the process of evaluating patients before surgery to 12 months after surgery.

## 3 Results

### 3.1 Baseline characteristics

**Fig. 2:**
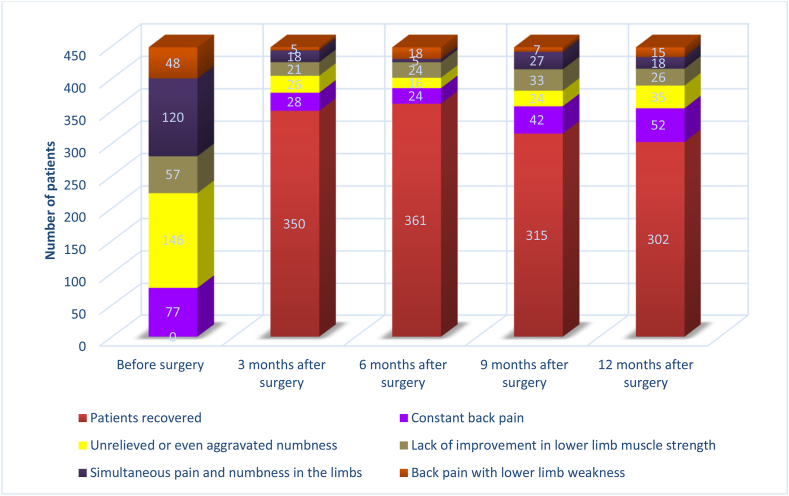
The recovery rate of patients up to 12 months after surgery.

A total of 512 patients meeting the criteria were enrolled. Initially, 37 patients were excluded due to hospital transfer, diagnosis of fractures, and malignancy. Subsequently, 475 patients underwent lumbar spine surgery. Another 27 patients were excluded due to undergoing surgery in two stages: pregnancy and lack of follow-up interest ….In the end, 302 patients were in the non-FBSS group, and 146 patients were in the FBSS group, with an incidence rate of 32.5 % (Fig. 1). The reasons for dissatisfaction became clearer through further follow-up: 52 people suffered from persistent lower back pain after surgery, 35 people experienced unrelieved or even aggravated numbness, 26 individuals reported a lack of improvement in lower limb muscle strength. 18 patients had simultaneous pain and numbness in their limbs, and 15 patients experienced lower back pain accompanied by weakness in the lower limbs (Fig. 2). In this study, 30 independent variables potentially influencing the occurrence of FBSS were examined. Based on the principle that the required sample size for multivariate analysis should be 5 to 10 times the number of independent variables [18], the size of the sample needed for this study was 150–300. Four hundred forty-eight patients were analyzed, exceeding the minimum sample size and meeting the statistical requirements.

Demographic data analysis showed that the mean age in the FBSS group was significantly higher compared to the healthy group (non-FBSS) (65.68 (8.66) vs. 62.62 (9.03), P < 0.05). Smoking was more prevalent in the FBSS group as a potential risk factor (61.6 % vs. 49.7 %, P < 0.05). No significant differences were observed between the two groups for other variables (P > 0.05) (Table 1).

**Table 1:** Evaluation of demographic information.

	FBSS	Non-FBSS	P-value^‡^
	(N = 146)	(N = 302)	
**Age**^**†**^**(yr),** mean (SD)	64.68 (8.66)	62.62 (9.03)	**0.022**
**Gender**∗**,** NO(%)			0.683
Male	76 (52.1 %)	151 (50.0 %)	
Female	70 (47.9 %)	151(50.0 %)	
**Height**^**†**^**(cm),** mean ± SD)	176.74 ± 8.74	175.02 ± 9.81	0.062
**Weight**^**†**^**(kg),** (mean ± SD)	88.25 ± 10.2	87.26 ± 8.99	0.298
**BMI(Kg/m**^**2**^**),** (mean ± SD)			
**Employment status**∗**,** NO(%) Unemployed Employed			0.08
	37 (25.3 %)	55 (18.2 %)	
	109 (74.7 %)	247 (81.8 %)	
**Smoking status**∗**,** NO(%)			**0.017**
Yes	90 (61.6 %)	150 (49.7 %)	
No	56 (38.4 %)	152 (50.3 %)	
**Opioid**∗**,** NO(%)			0.714
Yes	12 (8.2 %)	28 (9.3 %)	
No	134 (91.8 %)	274 (90.7 %)	
**Marital status**∗**,** NO(%)			0.739
Single	28 (19.2 %)	54 (17.9 %)	
Married	118 (80.8 %)	248 (82.1 %)	
**Level of education**∗**,** NO(%)			0.141
≤Diploma	97 (66.4 %)	221 (73.2 %)	
Callegiate	49 (33.6 %)	81 (26.8 %)	
**BMD**^**†**^**(T-score),** mean(SD)	−1.83 (0.44)	−1.85 (0.65)	0.779

∗:Chi-Square, †:*t*-test, **‡**: Statistical significant as p < 0.05. failed back surgery syndrome**)** FBSS(. body mass index (BMI), bone mineral density (BMD), standard deviation (SD).

### 3.2 Pre-surgery outcome

An analysis of pre-surgery factors revealed that repeated surgeries lead to a decrease in the success rate and an increased likelihood of developing FBSS. In other words, as the number of surgeries increases, the incidence of FBSS also rises (54.8 % vs. 35.5 %, P < 0.04). Additionally, the spread of symptoms to the lower limbs may be associated with the occurrence of FBSS. Therefore, patients who experienced leg pain or numbness in the lower limbs prior to surgery were at a higher risk of developing FBSS compared to other patients FBSS (50.7 % vs. 19.9 %, P < 0.05) (Table 2).

**Table 2:** Evaluation of pre-surgery factors and underlying diseases.

	FBSS	Non-FBSS	P-value^‡^
	(N = 146)	(N = 302)	
**Anxiety before surgery**^†^**,** mean (SD)	62.23 ± 12.4	63.60 ± 13.9	0.348
**Arthrose**∗**,** No(%)			0.096
Yes	37 (25.3 %)	56 (18.5 %)	
No	109 (74.7 %)	246 (81.5 %)	
**Rheumatoid Arthritis**∗**,** No(%)			0.130
Yes	35 (24.0 %)	(17.9 %)54	
No	(76.0 %)111	248 (82.1 %)	
**Diabetes**∗**,** No(%)			0.774
Yes	35 (23.97 %)	(23.2 %)70	
No	(76.02 %)111	231 (76.5 %)	
**Hypertension**∗**,** No(%)			0.373
Yes	41 (28.1 %)	73 (24.2 %)	
No	105 (71.9 %)	229 (75.8 %)	
**Number of surgeries** ∗**,** No(%)			**0.0001**
First time	66 (45.2 %)	195 (64.6 %)	
Recurrent	80 (54.8 %)	107 (35.5 %)	
**Pain location**∗**,** No(%)			**0.0001**
Low back pain without limb symptoms	72 (49.3 %)	242 (80.1 %)	
Unilateral limb symptom	21 (14.4 %)	28 (9.3 %)	
Bilateral limb symptom	53 (36.3 %)	32 (10.6 %)	
**Symptom duration (Month)**^†^**,** mean (SD)	11.3 (3.27)	11.8 (3.09)	0.178
**Claudication**∗**,** No(%)			0.353
Yes	21 (14.4 %)	54 (17.9 %)	
No	125 (85.6 %)	248 (82.1 %)	

∗: Chi-Square, †: *t*-test, **‡** : Statistical significant as p < 0.05. failed back surgery syndrome (FBSS), standard deviation (SD).

### 3.3 During surgery outcome

Analysis of intraoperative factors showed that the incidence of FBSS significantly varied among different surgical techniques. Therefore, the highest incidence of FBSS was observed in patients who underwent fixation with posterior lumbar interbody fusion (PLIF) (46.6 % vs. 13.9 %, P < 0.05) (Table 3).

**Table 3:** Evaluation of factors during surgery.

	FBSS	Non-FBSS	P-value^‡^
	(N = 146)	(N = 302)	
**Time of surgery**^†^**,** mean (SD)	120.8 (17.3)	117.5 (22.9)	0.076
**Type of complication**∗**,** No(%)			0.110
Laminectomy	15 (10.2 %)	27 (8.10 %)	
Spinal stenosis	19 (13.0 %)	63 (20.9 %)	
Spinal instability	25 (17.1 %)	53 (17.5 %)	
Spondylolisthesis	23 (15.7 %)	58 (19.2 %)	
Spondylolysis	36 (24.6 %)	46 (15.2 %)	
Multiple complications	28 (19.1 %)	55 (18.2 %)	
**Side Fixation**∗**,** No(%)			0.480
Unilateral	52 (35.6 %)	118 (39.07 %)	
Bilateral	94 (64.4 %)	184 (60.9 %)	
**Levels of surgery∗,** No(%)			0.296
Single level	70 (47.9 %)	129 (42.7 %)	
Multilevel	76 (52.1 %)	173 (57.3 %)	
**Type of Surgery**∗, No(%)			
Decompressio	25 (17.1 %)	164 (54.3 %)	**0.0001**
Fixation alone	53 (36.3 %)	96 (31.8 %)	
Fixation + PLIF	68 (46.6 %)	42 (13.9 %)	
**Sacrum fixation**∗**,** No(%)			0.138
Yes	36 (24.7 %)	95 (31.5 %)	
No	110 (75.3 %)	207 (68.5 %)	

∗:Chi-Square, †:*t*-test, **‡**: Statistical significant as p < 0.05. failed back surgery syndrome (FBSS), standard deviation (SD), posterior lumbar interbody fusion (PLIF).

### 3.4 Post-surgery outcome

The results of the study show that, Individuals with poor mental health post-surgery are at a higher risk of developing FBSS (70.03(12.6) vs. 40.8(12.1), P < 0.05). Additionally, the highest incidence of FBSS is found in individuals with poor quality of life (31.03(11.1) vs. 64.3 (16.4), P < 0.05). No significant differences were observed between the two groups in terms of rest time post-surgery, physiotherapy, and sleep quality (P > 0.05) (Table 4).

**Table 4:** Evaluation of factors post-surgery.

	FBSS	Non-FBSS	P-value^‡^
	(N = 146)	(N = 302)	
**Rest time(day)**^†^, mean (SD)	42.8 (12.6)	44.6 (11.6)	0.143
**Rehabilitation**∗, No(%)			0.420
Yes	49 (33.6 %)	90 (29.8 %)	
No	97 (66.4 %)	212 (70.2 %)	
**Psychopathy**^†^**,** mean (SD)	70.03 (12.6)	63.5 (12.2)	**0.0001**
**Quality of Life**^†^**,** mean (SD)	31.03 (11.1)	64.3 (16.4)	**0.0001**
**Sleep quality**^†^**,** mean (SD)	5.87 (1.71)	5.69 (2.15)	0.346

∗:Chi-Square, †:*t*-test, **‡**: Statistical significant as p < 0.05. failed back surgery syndrome (FBSS), standard deviation (SD).

was used to explain the strength and efficiency of the logistic regression model in distinguishing and classifying individuals with FBSS and non-FBSS. Overall, the classification accuracy of individuals by the fitted logistic regression model was 78.8 % (Table 5).

**Table 5:** Classification table.

		Predicted		Percentage Correct
		Non-FBSS	FBSS	
Observed	Non-FBSS	267	35	88.4
	FBSS	60	86	58.9
**Overall Percentage**				**78.8**

failed back surgery syndrome) FBSS(

Before determining the final model, the coding was done for qualitative variables. For each variable, one group was designated as the reference category to compare the odds of developing FBSS in other groups relative to the reference category (Table 6).

**Table 6:** Categorical variables codings.

		Frequency	Parameter coding	
			[1]	[2]
Type of surgery	Decompression	189	1.000	0.000
	Fixation	149	0.000	1.000
	Fixation + PLIF	110	0.000	0.0001
**Pain location**	Back-pain	314	1.000	0.0001
	Unilateral pain-limb	49	0.000	1.000
	Bilateral pain-limb	85	0.000	0.000
**Number of surgeries**	First time	261	1.000	
	Recurrent	187	0.000	
**Smoking status**	No	208	1.000	
	Yes	240	0.000	

posterior lumbar interbody fusion (PLIF).

Variables like smoking status and number of surgeries, which have two categories, receive scores of 0 and 1 as follows. The variable **Smoking status** [1] receives a score of 1 for non-smokers and a score of 0 for smokers. The score of 1 for the variable number of surgeries [1] pertains to individuals undergoing surgery for the first time, while a score of 0 is designated for those undergoing surgery more than once.

For variables with more than 2 categories, we choose a reference category and compare the others against it. The pain location [1] receives a score of 1 for individuals who experienced only back pain prior to surgery, and a score of 0 for those whose back pain radiated to one leg (Unilateral limb) or both legs (Bilateral limb) before the surgery.

The pain location [2] receives a score of 1 for individuals whose back pain radiated to one leg (Unilateral limb) before surgery, and a score of 0 for individuals who only had back pain (back pain) prior to surgery or for those whose back pain radiated to both legs (Bilateral limb).

The type of surgery variable [1] receives a score of 1 for individuals who undergo surgery using the decompression method, and a score of 0 for those who have undergone surgery using the fixation or fixation + PLIF methods. Additionally, the type of surgery variable [2] receives a score of 1 for individuals who undergo surgery using the Fixation method, and a score of 0 for those who have undergone surgery using the decompression or fixation + PLIF methods (Table 6).

Based on the odds ratio (OR), the selected surgical technique is the strongest factor in the occurrence of FBSS. Therefore, the likelihood of developing FBSS in individuals who underwent Fixation + PLIF surgery is 10.52 times greater than those who underwent decompression surgery. Additionally, patients who underwent Fixation + PLIF surgery are 2.83 times more likely to develop FBSS compared to those who underwent Fixation surgery. In our regression model, the second most influential factor in the occurrence of FBSS was radicular pain in the lower limbs. The likelihood of developing FBSS in individuals who had radicular pain in both lower limbs before surgery is 7.24 times higher than in those who only suffered from back pain. The likelihood of developing FBSS in individuals whose back pain radiated to both legs before surgery is 2.92 times higher than in those whose back pain radiated to only one leg. Smoking was identified as the third most influential factor in the occurrence of FBSS, with smokers having a 2.55 times higher likelihood of developing FBSS compared to non-smokers.

Based on the odds ratio (OR), the likelihood of developing FBSS in revision surgery is 2.50 times higher than in those undergoing surgery for the first time. The odds ratio for the variables of age and psychological disorders is reported to be 1.046 and 1.031, respectively. Thus, an increase in age and a higher score of psychological disorders significantly increase the likelihood of developing FBSS. The odds ratio for the quality of life variable is 0.967, indicating that an increase in the quality of life score significantly reduces the likelihood of developing FBSS (Table 7).

$$\ln\left(\frac{\hat{P}}{1-\hat{P}}\right)=\left(-\text{Beta}\times \text{Smoking}\;\text{status}\left(1\right)\right)+\left(-\text{Beta}\times \text{Number}\;\text{of}\;\text{surgeries}\;\left(1\right)\right)+\left(-\text{Beta}\times \text{Pain}\;\text{location}\;\left(1\right)\right)+\left(-\text{Beta}\times \text{Pain}\;\text{location}\;\left(2\right)\right)+\left(-\text{Beta}\times \text{type}\;\text{of}\;\text{surgery}\;\left(1\right)\right)+\left(-\text{Beta}\times \text{type}\;\text{of}\;\text{surgery}\;\left(2\right)\right)+\left(\text{Beta}\times \text{Age}\right)+\left(-\text{Beta}\times \text{Quality}\;\text{of}\;\text{life}\right)+\left(\text{Beta}\times \text{Psychopathy}\right)$$

$$\ln\left(\frac{\hat{P}}{1-\hat{P}}\right)=\left(-0.936\times \text{Smoking}\;\text{status}\left(1\right)\right)+\left(-0.918\times \text{Number}\;\text{of}\;\text{surgeries}\;\left(1\right)\right)+\left(-1.981\times \text{Pain}\;\text{location}\;\left(1\right)\right)+\left(-1.072\times \text{Pain}\;\text{location}\;\left(2\right)\right)+\left(-2.352\times \text{type}\;\text{of}\;\text{surgery}\;\left(1\right)\right)+\left(-1.041\times \text{type}\;\text{of}\;\text{surgery}\;\left(2\right)\right)+\left(0.045\times \text{Age}\right)+\left(-0.033\times \text{Quality}\;\text{of}\;\text{life}\right)+\left(0.031\times \text{Psychopathy}\right)$$

**Table 7:** Univariate logistic prediction model of FBSS.

Variables	Beta	S.E	Wald	Df	OR	95 % Confidence Interval for OR		P-value^‡^
						Lower	Upper	
**Type of surgery**			51.600	2				0.0001
**Type of surgery** [1]	−2.352	0.327	51.595	1	0.095	0.050	0.181	0.0001
**Type of surgery** [2]	−1.041	0.299	12.115	1	0.353	0.197	0.635	0.001
**Pain lo cation**			39.854	2				0.0001
**Pain location** [1]	−1.981	0.318	38.847	1	0.138	0.074	0.257	0.0001
**Pain location** [2]	−1.072	0.451	5.648	1	0.342	0.141	0.829	0.017
**Smoking status** [1]	−0.936	0.270	11.995	1	0.392	0.231	0.666	0.001
**Number of surgeries** [1]	−0.918	0.254	13.063	1	0.399	0.243	0.657	0.0001
**Age**	0.045	0.011	15.903	1	1.046	1.023	1.069	0.0001
**Psychopathy**	0.031	0.008	13.586	1	1.031	1.014	1.048	0.0001
**Quality of life**	−0.033	0.009	14.880	1	0.967	0.951	0.984	0.0001

Coefficient value (Beta), Standard error (S.E), Chi-square value (Wald), Degrees of freedom (Df), Odds ratio (OR). **‡**: Statistical significant as p < 0.05.

Finally, the logistic regression model was obtained as follows.

This model calculates the probability that an individual will develop FBSS.

**Fig. 3:**
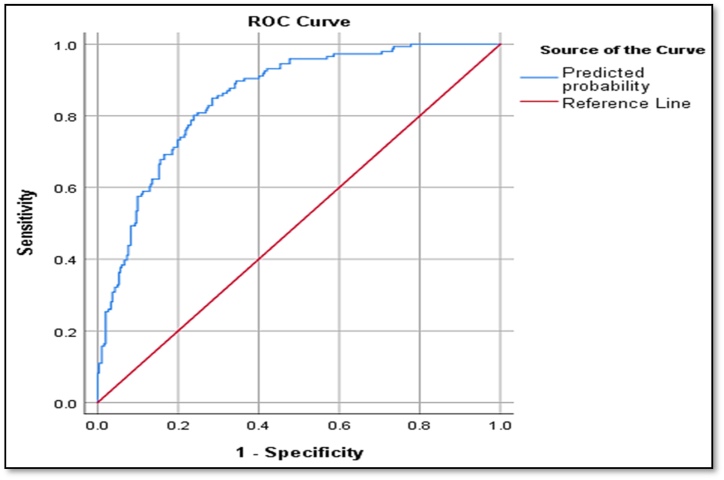
The area under the rock curve shows the performance of the logistic regression model in identifying and classifying people with FBSS or Non-FBSS. The x-axis is the sensitivity, which represents the possibility of predicting positive samples but actually negative samples. The y axis is 1-specific, which represents the possibility of predicting positive samples but actually positive samples. The blue real line is the ROC curve of the model, and the red line represents the ROC curve of random gues.

According to the results, the AUC (Area Under the ROC Curve) index is reported to be 0.852, indicating that the proposed logistic regression model has a high performance in correctly identifying and classifying individuals with FBSS and non-FBSS. The sensitivity of the prediction model is 58.9 %, and the specificity is 88.4 % (Table 8) (Fig. 3).

**Table 8:** Area under the ROC curve.

Predicted probability	Area	S.E	95 % Confidence Interval for Area		P-value
			Lower	Upper	
	0.852	0.018	0.816	0.887	0.0001

### 3.5 Application example of risk prediction model

Case Study1: Consider a non-smoking, 57-year-old married female patient with a BMI of 30.2 kg/m^2^. Six years ago, she underwent fixation surgery at the L3-L4 lumbar spine level. She presented with severe back pain and radicular pain in both lower limbs. Magnetic resonance imaging showed ASD (adjacent segment disease) at the L2-L3 vertebrae level. Additionally, flexion and extension radiographs revealed grade 3 spondylolisthesis at the L5-S1 vertebrae level. Thus, she underwent fixation surgery from the L2 to S1 vertebrae levels. Her surgery lasted 3 h and 45 min. Her quality of life and psychological disorder scores were 33 and 73, respectively. Now, we input these values into the logistic regression model.

$$\ln\left(\frac{\hat{P}}{1-\hat{P}}\right)=\left(-0.936\times 1\right)+\left(-0.918\times 0\right)+\left(-1.981\times 0\right)+\left(-1.072\times 0\right)+\left(-2.352\times 0\right)+\left(-1.041\times 1\right)+\left(0.045\times 57\right)+\left(-0.033\times 33\right)+\left(0.031\times 73\right)=1.762$$

$$\ln\left(\frac{\hat{P}}{1-\hat{P}}\right)=3.271\;e^{\ln\left(\frac{\hat{P}}{1-\hat{P}}\right)}=e^{1.762}\;\frac{\hat{P}}{1-\hat{P}}=5.824074\;\hat{P}=0.85$$

Not: e exponential = 2.718 (Napier’s Constant)

The predicted probability that this patient will develop FBSS is approximately 0.85 or 85 %. Since this probability is more significant than 0.5, according to the logistic regression model, this case is classified as an individual with FBSS.

Case Study 2: We consider a 63-year-old male patient who is a smoker, married, with a BMI of 28.5 kg/m^2^. This individual was a candidate for lumbar spine surgery for the second time due to recurrent disc herniation at the L3-L4 level and spondylolisthesis at the L4-L5 level. The reason for his visit was continuous pain in the lower back and left lower limb for 7 months, which had recently been accompanied by numbness in the left big toe. This patient underwent bilateral fixation + PLIF surgery, which lasted 2 h and 35 min. Following the surgery, he underwent 10 sessions of physiotherapy, and his quality of life and psychological disturbance scores were reported as 25 and 68, respectively. Now we will input the values into the following logistic regression model.

$$\ln\left(\frac{\hat{P}}{1-\hat{P}}\right)=\left(-0.936\times 0\right)+\left(-0.918\times 0\right)+\left(-1.981\times 0\right)+\left(-1.072\times 1\right)+\left(-2.352\times 0\right)+\left(-1.041\times 0\right)+\left(0.045\times 63\right)+\left(-0.033\times 25\right)+\left(0.031\times 68\right)=3.271$$

$$\ln\left(\frac{\hat{P}}{1-\hat{P}}\right)=3.271\;e^{\ln\left(\frac{\hat{P}}{1-\hat{P}}\right)}=e^{3.271}\;\frac{\hat{P}}{1-\hat{P}}=26.33766\;\hat{P}=0.96$$

Not: e exponential = 2.718 (Napier’s Constant)

The predicted probability for this case to be diagnosed with FBSS is approximately 0.96, or in other words, 96 %. Since this probability is greater than 0.5, according to the logistic regression model, we classify this case as belonging to the group of individuals with FBSS.

## 4 Discussion

The present study aims to analyze the risk factors and develop a risk prediction model for FBSS. A risk prediction model was adopted, predicting logistic regression outcomes based on clinical and medical data. In this study, the surgical technique was identified as the strongest factor influencing the occurrence of FBSS. The highest incidence of FBSS was first observed in the Fixation + PLIF group, followed by the Fixation group. Recent reports indicate that pedicle screw fixation techniques, bilateral muscle dissection, removal of posterior elements, and loss of bony structure can lead to spinal instability and pain of unknown origin [19,20]. According to Souslian & Patel (2024), after PLIF surgery, the potential for load distribution among spinal structures increases, which may result in axial pain [21]. Cicek et al. (2017) reported similar findings [22]. In patients undergoing PLIF + Fixation surgery, the entire bilateral facet joints are removed, the disc is completely evacuated, and a relatively small intervertebral cage acts as a focal pivot point. Thus, despite pedicle screw fixation, the structure may inherently be unstable [23]. However, contrary to these findings, some studies have shown no significant difference between spinal fixation techniques with and without intervertebral fusion [24]. The discrepancy in results may be due to variations in postoperative follow-up duration, levels of spinal fixation, and different surgical techniques.

Data analysis indicates that radicular pain in the lower limbs before surgery is the second most influential factor in FBSS. Supporting these findings, Wenbo et al. (2022) argue that preoperative radicular pain in the lower limbs is a significant factor in the development of FBSS and should be investigated as a marker for identifying at-risk populations [25]. Evandro et al. (2020) reported similar results [2]. In contrast to these findings, some reports suggest that surgical outcomes may not be significantly related to the type of preoperative pain or its severity [1]. The discrepancy in results may be influenced by the duration of symptoms before surgery and the criteria for patient inclusion. The optimal timing for decompression surgery remains unclear. Historically, early surgical intervention for symptomatic spinal stenosis has been recommended, based on the view that the condition is always progressive. Unlike peripheral nerves, nerve roots lack a blood-nerve barrier, and prolonged compressive lesions lead to intraneural edema. Over time, this edema causes nerve fibrosis, a process that is irreversible even with surgical intervention [26]. Additionally, research has shown that long-term symptomatic radiculopathy is affected by multiple damaging factors and is more complex than simple neural dysfunction caused by physical pressure [27].

Data analysis suggests that smoking is one of the significant risk factors for the development of FBSS. In this context, a study by Mekhail et al. (2020) with a one-year follow-up found that the incidence of FBSS was significantly higher in current smokers compared to non-smokers and those who had quit smoking [28]. Robson et al. (2019) reported similar findings [23]. A meta-analysis indicated that individuals who quit smoking three months before surgery were less likely to develop FBSS compared to smokers [29]. In contrast to these results, some studies did not find a significant difference in the incidence of FBSS between smokers and non-smokers [30]. The discrepancy in results may be due to the type of intervention, the extent of iatrogenic tissue damage, and the sample size of the studies. Generally, nicotine destroys vascular endothelium, playing a crucial role in vasoconstriction, blood flow disruption, synthesis and secretion of biologically active factors, neovascularization, and immune responses. After spinal surgery, the circulatory system plays a key role in tissue healing and postoperative recovery. Therefore, when the vascular endothelium is damaged, the post-surgical healing process becomes disrupted and inefficient in smokers. The more extensive the iatrogenic soft tissue damage during surgery, the more postoperative complications are likely to increase. These findings are supported by several studies reporting increased inflammatory markers in smokers [31,32].

Findings from the study indicate that revision surgery is a potential risk factor for developing FBSS. Therefore, patients with previous lumbar surgery are at higher risk. Researchers believe that the number of previous spinal surgeries is a significant predictor of surgical outcomes [33]. Montenegro et al. (2021) found that the average functional status score of 46 % of patients significantly decreased six months after revision surgery [34]. Moaven et al. (2020) stated that revision surgery is much more complicated than primary surgery due to unclear anatomical levels and scars around the nerves. This complexity requires a high skill level [33]. In revision surgeries, the absence of spinous processes, bony structures, and fibrous tissue growth in the surgical area leads to significant anatomical changes. Adhesions in the surgical area increase the risk of nerve element damage and dural tears. Therefore, there is concern that the success rate of the second surgery is lower than the first. Researchers have noted that the incidence of FBSS increases from 8 % in primary surgeries to 48 % in revision surgeries. However, the degree of difference may vary depending on surgical techniques and the type of condition [35].

Data analysis indicates that age is one of the risk factors for patients undergoing spinal surgery. Early studies have identified age as an important underlying factor in the occurrence of FBSS and believe it requires special consideration when selecting treatment options [35]. Wenbo et al. (2022) argue that older patients experience a significant increase in postoperative complications and often require revision surgery [25]. Other studies in this field have reported similar findings [3,36]. Changes in pain perception due to aging affect the pain experience in various ways, and some of these effects are still unknown. These assumptions are based on anatomical changes in older individuals, such as narrowing of the spinal canal and foramina, increased pressure on neural elements, and disruption of microcirculation, which exponentially decrease the success rate after surgery. Researchers have found that loss of lumbar lordosis, increased thoracic kyphosis, and stiffening of the ligamentum flavum are frequent changes that contribute to spinal degeneration and are common sources of back pain after surgery in older individuals [37].

Data from the study suggest that specific psychological factors, including significant levels of depression, anxiety, phobic anxiety, paranoid ideation, psychosis, obsessive-compulsive disorder, and somatic complaints, lead to an increased incidence of FBSS. Recent reports indicate that these psychological factors affect individual changes in pain sensitivity, thereby influencing pain perception [13,38]. The study by Sebaaly et al. (2018) showed a bidirectional relationship between pain and depression [39]. Elsamadicy et al. (2018) believe that mental health is a much stronger predictor of disability from back pain than structural abnormalities [3]. A recent study identified depression as a risk factor for experiencing postoperative pain in specific areas, including the head, neck/shoulder, and back [7]. In contrast to these findings, other studies showed no significant difference between psychological disorders and postoperative pain with a one-year follow-up [40]. The discrepancy in results may be due to the type of tools used to measure mental health and the duration of follow-up after surgery. In short-term follow-up, it may be difficult to differentiate between nonspecific pain sources such as skin incision, iatrogenic tissue damage, reactive spasms, and nerve root inflammation. Additionally, pain may also originate from the central nervous system. In this context, the results of a study by Graham et al. with a five-year follow-up reported similar findings, illustrating this issue well [20].

The existing literature indicates that psychological disorders can result from stressors and disrupt behavioral patterns. In other words, individuals who experience pain due to their spinal condition often avoid normal work and recreational activities, significantly reducing their quality of life [41]. As noted in the present study, poor quality of life predicts the occurrence of FBSS. Inoue et al. (2017) found that an increased incidence of FBSS is associated with low quality of life and high levels of functional disability [40]. Therefore, psychological disorders can elevate the prevalence of FBSS both directly and indirectly. However, it is important to note that awareness of these factors should not prevent patients from undergoing spinal surgery when significant pathology and surgical indications are present. Instead, these risk factors necessitate careful consideration and optimization of surgical timing. Patients with higher surgical risk in spinal surgery may achieve better outcomes, particularly when interventions are prompt. This is because prolonged pain and discomfort in this population can exacerbate existing psychosocial stressors and negatively impact the success rate of surgery.

It is logical that "the most effective treatment for FBSS is to prevent FBSS itself," as aging and the exacerbation of psychological, physical, and mechanical effects experienced by patients transform it into a multifactorial, complex, and painful syndrome. The detrimental effects of FBSS are well known, and current strategies and treatment methods are only available after the onset of the disease. Furthermore, most patients with FBSS experience varying degrees of disability, which brings significant anxiety and economic burdens to them and their families. Therefore, early screening for this condition is of particular importance. The prediction model presented in the current study demonstrated good performance and is easy to apply in clinical practice. This model may be useful for preventing and treating FBSS, as well as identifying at-risk populations in clinical settings.

### 4.1 Limitations

There are several limitations to this study. First, a relatively short follow-up period may obscure changes in outcome indicators due to other degenerations. Additionally, the patient's risk factors examined in this study were self-reported. This introduces potential recall bias and subjective interpretations. Future research should examine a wider range of clinical variables to enhance the comprehensiveness and completeness of the information obtained by the prediction model. Moreover, using a multicenter approach for sample collection would increase the model's validity and generalizability.

## 5 Conclusion

In summary, based on the results of multivariate logistic regression analysis, this study demonstrated that the selected surgical technique, preoperative pain symptoms, smoking, revision surgery, age, mental health, and quality of life are risk factors for FBSS. Increased awareness in this area can serve as a tool for physicians to identify at-risk populations and provide more effective management to reduce discrepancies between patient and physician expectations. Relying on these factors, an initial FBSS risk prediction model was developed. Additionally, the ROC curve indicated that this logistic regression model is effective in accurately identifying and classifying individuals.

## CRediT authorship contribution statement

**Parisa Hajilo:** Project administration, Visualization. **Behzad Imani:** Conceptualization, Investigation, Writing – review & editing. **Shirdel Zandi:** Data curation, Investigation, Writing – original draft. **Ali Mehrafshan:** Validation, Funding acquisition, Resources, Methodology. **Salman khazaei:** Software, Formal analysis, Validation.

## Ethics approval

This study was reviewed and approved by Ethics Committee of Hamedan University of Medical Sciences with the approval number: IR.UMSHA.REC.1402.553.

## Availability of data and materials

Data associated with the study has not been deposited into a publicly available repository. Data are available from the corresponding author on reasonable request.

## Funding

The study was funded by Vice-chancellor for Research and Technology, 10.13039/501100004697Hamadan University of Medical Sciences (140208237083).

## Declaration of competing interest

The authors declare that they have no known competing financial interests or personal relationships that could have appeared to influence the work reported in this paper.
